# Asians Have Higher Risk of Developing Pancreatic Necrosis in Inflammatory Bowel Disease Patient Population: A National Inpatient Sample Database Study

**DOI:** 10.7759/cureus.10573

**Published:** 2020-09-21

**Authors:** Praneeth Kudaravalli, Nishant Tripathi, Olalekan Akanbi, Pradeep Yarra, Marwan Abougergi

**Affiliations:** 1 Hospital Medicine, University of Kentucky, Lexington, USA; 2 Internal Medicine/Gastroenterology, University of South Carolina School of Medicine, Columbia, USA

**Keywords:** acute pancreatitis, national inpatient sample, inflammatory bowel disease

## Abstract

Background

The aim of this study is to evaluate race-associated risk factors of acute pancreatitis (AP) in inflammatory bowel disease (IBD) patients.

Methods

A retrospective analysis using 2016 and 2017 National Inpatient Sample database was performed. Inclusion criteria were principal diagnosis of AP and a secondary diagnosis of IBD. Patients below 18 years of age were excluded. The primary outcome was in-hospital mortality rate and secondary outcomes included pancreatic necrosis, surgical necrosectomy, total hospitalization charges, total parenteral nutrition use, and length of stay. For the primary and secondary outcomes, adjusted odds ratios (aORs) and mean difference calculation using multivariate regression were calculated.

Results

A total of 7,060 patients with AP in IBD were identified; of which 53.5% were female. The use of Medicaid was significantly higher in blacks (39.5%), Hispanics (32.6%), and Asian/Pacific Islanders (40%) compared to whites (19.9%). Approximately 63.2% of AP patients in IBD received care at an urban teaching hospital. Pancreatic necrosis was noted to be highest in Asians or Pacific Islanders compared to whites (aOR 12.62, 95% CI 1.00-159.3, p = 0.05).

Conclusion

Our study shows that racial disparities exist among AP in IBD patients with pancreatic necrosis being more common in Asians and Pacific Islanders compared to whites. Identification of potential causes of these disparities is of paramount importance to expand access to healthcare.

## Introduction

Acute pancreatitis (AP) has emerged as the most common gastrointestinal diagnosis at discharge [1]. AP-related inpatient hospitalizations are associated with an estimated annual cost of 2.6 billion dollars. A recently published meta-analysis estimated the AP global incidence and mortality to be 33.74 per 100,000 person-years and 1.60 deaths per 100,000 person-years, respectively [2]. The incidence of AP has been rising over the last several years [3,4]. Similarly, a recent Global Burden of Disease analysis identified an increase in global crude prevalence of inflammatory bowel disease (IBD) from 686 cases per million in 1990 to 896 cases per million in 2017 [5]. A population-based cohort study in Taiwan [6] determined the overall incidence of AP in patients with IBD to be 3.56 times higher than that of the general population. Such a finding was likely secondary to IBD itself and associated treatments [7]. A 16-year Danish nationwide follow-up study documented an elevated risk of AP in both forms of IBD: Crohn’s disease (CD) and ulcerative colitis (UC) [8].

Moreover, length of stay and cost of hospitalization were significantly higher among patients with AP and concomitant IBD than in patients without IBD [9]. While there has been a two- to threefold increased risk of pancreatitis in blacks compared to whites, racial disparities in AP, with or without IBD, have not been adequately studied [4]. Prevalence of lifestyle factors, namely heavy alcohol and tobacco consumption, is similar in both races and therefore do not explain such a significant racial disparity in AP [10]. A retrospective study performed in California [11] between 1996 and 2009 identified the IBD prevalence among various racial-ethnic groups as follows: 14.9% blacks, 15.4% Hispanic, 22.4% Asian, and 46.1% whites. Multiple studies have documented the increasing prevalence of IBD in the non-white population outside of the United States [12-15]. Based on the Simple Clinical Colitis Activity Index, Asians and Hispanics appeared to present with more severe UC compared to whites [16]. In patients with CD, blacks experienced a higher incidence of arthritis and uveitis compared to whites. On the other hand, among patients with UC, whites experienced a higher incidence of joint symptoms and osteoporosis compared to Hispanics [17].

To the best of our knowledge, no previous study has evaluated race-associated risk factors for AP in patients with IBD. Racial disparities are barriers to optimal healthcare delivery, but whether race influences the incidence, prevalence, or severity of AP in patients with IBD is yet undetermined. However, uncovering the implications of race in AP pathogenesis in patients with concomitant IBD, as well as ascertaining etiologies (e.g. genetics, diet, geography, etc.) behind such disparities is of paramount importance.

This study seeks to determine whether a racial disparity exists for AP in patients with IBD. Findings from this study will help identify high-risk patients, which will ultimately help public health efforts not only in disease prevention, but also its optimal management.

## Materials and methods

Data source and patient selection

This was a retrospective cohort study utilizing the Agency for Healthcare Research and Quality’s Healthcare Cost and Utilization Project National Inpatient Sample (NIS) for the years 2016 and 2017. The NIS is the largest publicly available all-payer in-hospital care database in the United States [18].

In 2017, the database contained 7.1 million hospital stays from 4,585 hospitals across 48 states. It is designed as a stratified probability sample to be representative of all nonfederal acute care inpatient hospitalizations nationwide. Briefly, hospitals are stratified according to ownership/control, number of beds, teaching status, urban/rural location, and geographic region. A 20% probability sample of all hospitalizations within each stratum is then collected. Those hospital stays are recorded and information about patient demographics; principal and secondary diagnoses; vital status at discharge; readmission; and resource use, including length of stay, procedures performed, and total hospitalization costs and charges, is entered into the NIS. Each discharge is then weighted (weight = total number of discharges from all acute care hospitals in the United States divided by the number of discharges included in nationwide readmissions database) to make the NIS nationally representative. The NIS contains both patient- and hospital-level information. Up to 40 discharge diagnoses and 25 procedures are collected for each patient using the International Classification of Diseases, 10th Revision, Clinical Modification (ICD-10 CM). The NIS has been previously used to provide reliable estimates of the burden of gastrointestinal diseases [19].

Study population

Patients were included in the study if they had (1) a principal diagnosis of AP and (2) a secondary diagnosis of either CD or UC. The following ICD-10 CM codes were used: AP: K85.xx, CD: K50.xx, UC: K51.xx. Patients were excluded if they were younger than 18 years. The institutional review board of University of Kentucky deemed the research project exempt from approval because it is a retrospective review of already collected, de-identified data.

Study outcomes

The primary outcome was in-hospital all-cause mortality. The secondary outcomes were (a) resource utilization: length of stay and total hospitalization costs and charges; (b) proportion of necrosis and infected necrosis; and (c) percentage of total parenteral nutrition (TPN), necrosectomy, and radiology-guided drain placement. The exposure of interest was race, divided into four subgroups: whites, blacks, Hispanic, and Asian or Pacific Islander. 

Definition of variables

We used NIS variables to identify each patient’s age (in years), sex, median household income for patient’s zip code [(1) $1-$42,999; (2) $43,000-$53,999; (3) $54,000-$70,999; and (4) $71,000 or more], primary expected payer (Medicare, Medicaid, private insurance, and uninsured), hospital size based on number of beds (small, medium, and large), urban location, and teaching status. For the in-hospital mortality rate, the patient’s vital status at discharge, which is directly coded in the NIS, was used. Length of hospital stay and total hospitalization charges are variables directly coded in the NIS. Total hospital charges represent the amount that the hospitals billed for the entire hospital stay but do not reflect the actual cost of care. The Healthcare Cost and Utilization Project provides data that contain hospital-specific cost-to-charge ratios based on all-payer inpatient cost. This cost information is obtained from the hospital accounting reports collected by the Centers for Medicare and Medicaid Services [20]. Using this information, total hospitalization costs were calculated by multiplying total hospital charges by the corresponding cost-to-charge ratio. The presence of necrosis and infected necrosis was identified using the ICD-10 CM codes I85.x1 and I85.x2, respectively. TPN use was identified using the 3E0436Z code; endoscopic necrosectomy using the codes 0F9G8ZZ and 0FBG8ZZ; surgical necrosectomy using codes 0FBG0ZZ,0FBG4ZZ,0F9G0ZZ, and 0F9G40Z; and radiology-guided drain placement using the code 0F9G30Z.

Statistical analyses

All data analyses were conducted using STATA, version 13.0 (StataCorp, College Station, TX). NIS is based on a complex sampling design that includes stratification, clustering, and weighting. This software facilitates analysis to produce nationally representative unbiased results, variance estimates, and p-values. Weighting of patient-level observations was implemented to obtain estimates for the entire population of hospitalized patients with IBD in the United States. Proportions were compared with the Fisher exact test, while continuous variables were compared using the Student t-test. Univariate linear (continuous outcomes) or logistic (dichotomous outcomes) regression analysis was used to calculate unadjusted odds ratios for the primary and secondary outcomes. Subsequently, multivariate regression analysis was used to adjust the results for potential confounders. Multivariable regression models were built by including all confounders that were significantly associated with the outcome on the univariate analysis with a cutoff p-value of 0.2. All statistical analyses were two-sided, with a cutoff p-value of 0.05.

## Results

Between 2016 and 2017, 7,060 patients with IBD were admitted to the hospital for AP, of which 53.5% were female. The prevalence of AP among patients hospitalized with IBD is highest among whites at 79%. Use of Medicaid was significantly higher in non-whites compared to whites (39.5% in blacks, 32.6% in Hispanics, and 40% in Asian or Pacific Islanders versus 19.9% in whites, p = 0.004) The highest median income was made by Asians or Pacific Islanders, with 66.7% of the population earning $74,000 or more. Urban teaching hospitals were the most common setting for whites, blacks, Hispanics, and Asians or Pacific Islanders, including 61.7%, 66.5%, 72.7%, and 66.7% of patients, respectively. The southern region was the most common location, comprising 37.6% of patients, while no statistical significance was noted regarding hospital bed size. These results are shown in Table 1.

**Table 1:** Patient population characteristics. HMO, health maintenance organization

	White	Black	Hispanic	Asian or Pacific Islander	Total	P-value
Acute pancreatitis (%)	79	12.3	7.4	1.1		
Age (mean)	49	43.7	40.8	49.8		
Females (%)	53.5	51.8	58.6	33.3	53.5	0.3
Insurance status (%)						0.004
Medicare	35.1	25	18.6	20	32.5	
Medicaid	19.9	39.5	32.6	40	23.4	
Private + HMO	41.2	27.6	46.5	40	39.9	
Self-pay	3.7	7.9	2.3	0	4.1	
Median income (%)						0
$1-$43,999	22.3	52.5	26.8	0	26.1	
$44,000-$55,999	27.7	16.8	26.8	6.7	26.1	
$56,000-$73,999	26.6	21.2	26.8	26.7	25.9	
$74,000 or more	23.3	9.3	19.5	66.7	21.8	
Location/teaching status of hospital (%)						0.02
Rural	12	7.3	1	0	10.5	
Urban non-teaching	26.2	26.2	26.2	33.3	26.3	
Urban teaching	61.7	66.5	72.7	66.7	63.2	
Region of hospital (%)						0
Northeast	22.1	9.8	19.2	33.3	20.5	
Midwest	25.9	22.6	17.2	13.3	24.7	
South	35.2	57.3	33.3	13.3	37.6	
West	16.8	10.4	30.3	40	17.3	
Hospital bed size (%)						0.2
Small	20.9	19.5	22.2	26.7	21	
Medium	31.5	24.4	25.2	46.7	30.3	
Large	47.5	56.1	52.5	26.7	48.7	

Multivariate regression analysis for adjusted odds ratios (aOR) and mean difference was performed, which showed pancreatic necrosis to be more frequent in Asians or Pacific Islanders compared to the white patient population (aOR 12.62, 95% CI 0.99-159.3, p = 0.05). None of the patients had a radiology-guided drain placement. In-hospital mortality and necrosectomy had insufficient outcomes to be analyzed, while other variables including TPN use, total hospitalization charges, total hospitalization costs, and length of stay had no statistical significance (Table 2).

**Table 2:** Multivariate regression analysis for aOR and mean difference calculation (race compared to whites). aOR, adjusted odds ratio; TPN, total parenteral nutrition

Variables	aOR (95% CI)	P-value
Pancreatic necrosis		
Black	3.23 (0.77 to 13.59)	0.11
Hispanic	4.13 (0.99 to 17.26)	0.052
Asian or Pacific Islander	12.62 (1.00 to 159.3)	0.05
TPN use		
Black	9.48 (0.84 to 115.37)	0.06
Hispanic	6.08 (0.36 to 100.77)	0.2
Asian or Pacific Islander		
Total hospitalization charges		
Black	-1357.77 (-12169.68 to 9454.14)	0.8
Hispanic	18331.93 (-17884.3 to 54548.16)	0.32
Asian or Pacific Islander	-8615.512 (-52415.31 to 35184.28)	0.7
Total hospitalization costs		
Black	92.69 (-2329.54 to 2514.93)	0.08
Hispanic	2150.65 (-3734.69 to 8036.005)	0.72
Asian or Pacific Islander	1758.13 (-7945.377 to 11461.64)	0.36
Length of stay		
Black	-0.26 (-1.14 to 0.60)	0.55
Hispanic	-0.11 (-1.28 to 1.06)	0.86
Asian or Pacific Islander	-0.42 (-3.26 to 2.43)	0.77

## Discussion

Our study aimed to identify any racial disparities in individuals hospitalized with AP among the IBD patient population. Our results showed that there is a clear racial disparity between Asian and Pacific Islanders compared to whites in the prevalence of pancreatic necrosis. 

Various factors have been identified that trigger AP and complicate the course of IBD. A recent study by Ramos et al. [21] showed the most common causes of AP are thiopurines and gallstones. Thiopurine-induced AP is usually uncomplicated and self-limited, and new genetic markers are being developed to identify thiopurine-induced AP, which may prove to be a useful tool in the future when determining which patients will benefit from this therapy. Exocrine dysfunction and pancreatic duct abnormalities have also been identified in up to 18% of asymptomatic patients with IBD [22]. The clinical significance of exocrine dysfunction is yet to be determined even though it is the most common pancreatic manifestation in IBD patient population. Autoimmune pancreatitis (AIP) should be among the differential diagnoses for AP, as 27% of patients with AIP also have IBD, in particular UC [21].

A study by Fagenholz et al. [4] in 2007 showed increasing rates of AP-related hospitalizations in the United States, with higher rates in blacks compared to whites. Similarly, there is an increasing prevalence of IBD in the non-white population [12,14,15]. Our study is unique in that it helped identify racial disparities of AP in the IBD patient population. Asians and Pacific Islanders were found to be at a higher risk of developing pancreatic necrosis compared to whites (aOR 12.62, 95% CI 1.00-159.3, p = 0.05).

Pancreatic necrosis is a challenging clinical problem that occurs in 15%-25% of patients with AP, and the mortality rates remain high, especially when infected [23]. Pancreatic necrosis in AP represents the most severe disease form and is often associated with multisystem organ failure. An extent of tissue necrosis >30% is related to organ failure [24]. Infection remains a major complication of pancreatic necrosis; however, the use of prophylactic antibiotics has not shown a reduction in infection of pancreatic necrosis or mortality [25]. As patients with pancreatic necrosis often have an increased risk of complications during their hospital stay, the early diagnosis of necrotizing pancreatitis is of paramount importance [26]. Contrast-enhanced CT (CECT) remains the modality of choice for the diagnosis and quantification of pancreatic necrosis [27]. Pancreatic necrosis usually develops in the first 48 hours of AP, and CT performed in the first 12 hours of symptoms onset can be falsely negative [28]. CECT is usually recommended 72 hours after symptom onset as it accurately identifies tissue necrosis >30% [27,29,30]. If AP does not respond to conservative management, early imaging might be prudent in Asians or Pacific Islanders to evaluate for the presence of pancreatic necrosis. Not enough is known about the frequency of pancreatic necrosis in patients with IBD; however, corticosteroids used for acute IBD flares may increase the risk of necrosis and infection of pancreatic fluid collection [21]. In case of active IBD in patients with AP, biologics, preferably infliximab, should be considered over steroids.

This wide spectrum of manifestations complicates the care of IBD, and a collaborative approach with a pancreas specialist for additional workup to determine underlying etiology and define the follow-up of these patients is required for optimal care. 

Our study has several limitations. The retrospective design confers an inherent limitation. Although we were able to identify statistically significant results, our sample size was not large enough for a precise confidence interval measurement. As the NIS database does not have data on medications, we could not control for that variable. In addition, we could not separate CD from UC to better understand the racial differences between these two subgroups. These discrepancies should be further evaluated with a larger sample size in future studies. Further understanding will help identify at-risk populations and will eventually lead to improved care to ultimately reduce the incidence and prevalence of AP in IBD patients. 

## Conclusions

Our study is unique in that it provides insights into racial disparity that exists for pancreatic necrosis in patients with underlying IBD. Identification of potential causes of these disparities is of paramount importance to expand access to healthcare regardless of race. This complicates the care of both IBD and AP, thus requiring targeted interventions to at-risk groups as well as a collaborative approach for the best outcomes.
